# Intrapericardial Diaphragmatic Hernia Following the Convergence Procedure for Atrial Fibrillation

**DOI:** 10.7759/cureus.51484

**Published:** 2024-01-01

**Authors:** Muhammad Almas Baig, Muhammad Haseeb ul Rasool, Arslan Ahmad, Daniel Miller, Hazem Abosheaishaa, Muhammad Burhan Majeed Rana

**Affiliations:** 1 Internal Medicine, Icahn School of Medicine at Mount Sinai, Queens Hospital Center, New York, USA; 2 Medicine, Icahn School of Medicine at Mount Sinai, Queens Hospital Center, New York, USA; 3 Cardiology, Oklahoma Heart Hospital South, Oklahoma City, USA; 4 Internal Medicine/Gastroenterology, Cairo University, Cairo, EGY

**Keywords:** robotic surgery, atrial fibrillation, diaphragmatic hernia, convergence procedure, pericardial hernia

## Abstract

This case report highlights a very rare variant of diaphragmatic hernia, namely, an intrapericardial diaphragmatic hernia, which can arise as a complication of the convergence procedure. A 77-year-old man, presenting with chronic shortness of breath and fatigue, was unexpectedly found to have herniation of the transverse colon into the pericardial cavity through a diaphragmatic-pericardial defect. The diaphragmatic defect was repaired with mesh via robotic surgery. The patient reported resolution of his symptoms at the six-month follow-up.

## Introduction

The convergence procedure is becoming a standard therapy for long-standing and persistent atrial fibrillation. The initial, surgical portion of this procedure involves a trans-diaphragmatic approach and opening of the pericardium to reach the epicardial surface of the heart [1,2]. Subsequently, the pericardium is typically not closed, which leaves a defect that carries the potential for the development of a diaphragmatic hernia into the pericardial space. These can include intra-pericardial diaphragmatic hernia (IPDH), trans-diaphragmatic pericardial hernia (TDPH), or peritoneal-pericardial diaphragmatic hernia (PPDH) [3-5].

IPDH can result from an acquired or congenital diaphragmatic defect of the central tendon [3]. Acquired intra-pericardial diaphragmatic hernia is rare and usually secondary to a prior iatrogenic creation of a trans-diaphragmatic pericardial defect [6-10].

## Case presentation

A 77-year-old male was experiencing unexplained, refractory shortness of breath, exercise intolerance, and fatigue. During the work-up, he was incidentally found to have herniation of the transverse colon through a pericardial-diaphragmatic defect into the epicardial fat, three years after undergoing a convergent procedure for atrial fibrillation.

The patient had a past cardiac history of sick sinus syndrome status post (s/p) dual chamber permanent pacemaker placement (PPM), coronary artery disease s/p percutaneous coronary intervention (PCI) of the Left Anterior Descending artery, mild-moderate aortic regurgitation, diastolic heart failure/NYHA class II with LVEF 55-60%, mild carotid artery stenosis, and peripheral vascular disease with 75% stenosis of the proximal celiac, superior mesenteric artery (SMA), and inferior mesenteric artery (IMA). His non-cardiac medical history included psoriatic arthritis, obstructive sleep apnea, tracheomalacia, prostate cancer, lower back pain, and lumbar spondylosis. Furthermore, his surgical history included right shoulder surgery, bladder surgery, corneal surgery, Nissen fundoplication surgery for GERD, and left knee replacement.

The patient was diagnosed with paroxysmal atrial fibrillation (AF), and anti-arrhythmic medications never seemed to alleviate symptoms. He continued to experience AF events. His primary symptoms were shortness of breath and occasional palpitations, which worsened over time. His CHA2DS2-VaSc score was 3 (hypertension, age, and vascular disease), and he could not be fully anticoagulated due to a history of lower gastrointestinal bleeding and hematuria. The last interrogation of his pacemaker revealed an AF burden of 15% prior to the convergence procedure. The surgical portion of the convergence procedure was then performed via a diaphragmatic approach. One month later, he had an episode of slurred speech which led to endoscopic left atrial appendage closure (LAAC) therapy. The electrophysiologist portion of the convergence procedure was performed subsequently. During subsequent visits, his AF symptoms seemed to improve, and the AF burden was reduced to 1%. However, he still had some residual shortness of breath, which was thought to be secondary to his tracheobronchomalacia. Two years later, he began experiencing worsening shortness of breath and exercise tolerance, and occasional chest pains. Thus, he underwent an extensive workup, including an echocardiogram, stress tests, coronary angiograms, pulmonary function test (PFT), computed tomography (CT), and bronchoscopy. However, nothing significant was found, except for a decline in diffusing capacity for carbon monoxide (DLCO) and mild ground-glass and reticular changes in the bilateral bases of the lungs. A CT angiogram of the chest revealed intrapericardial herniation of a segment of the transverse colon.

**Figure 1:**
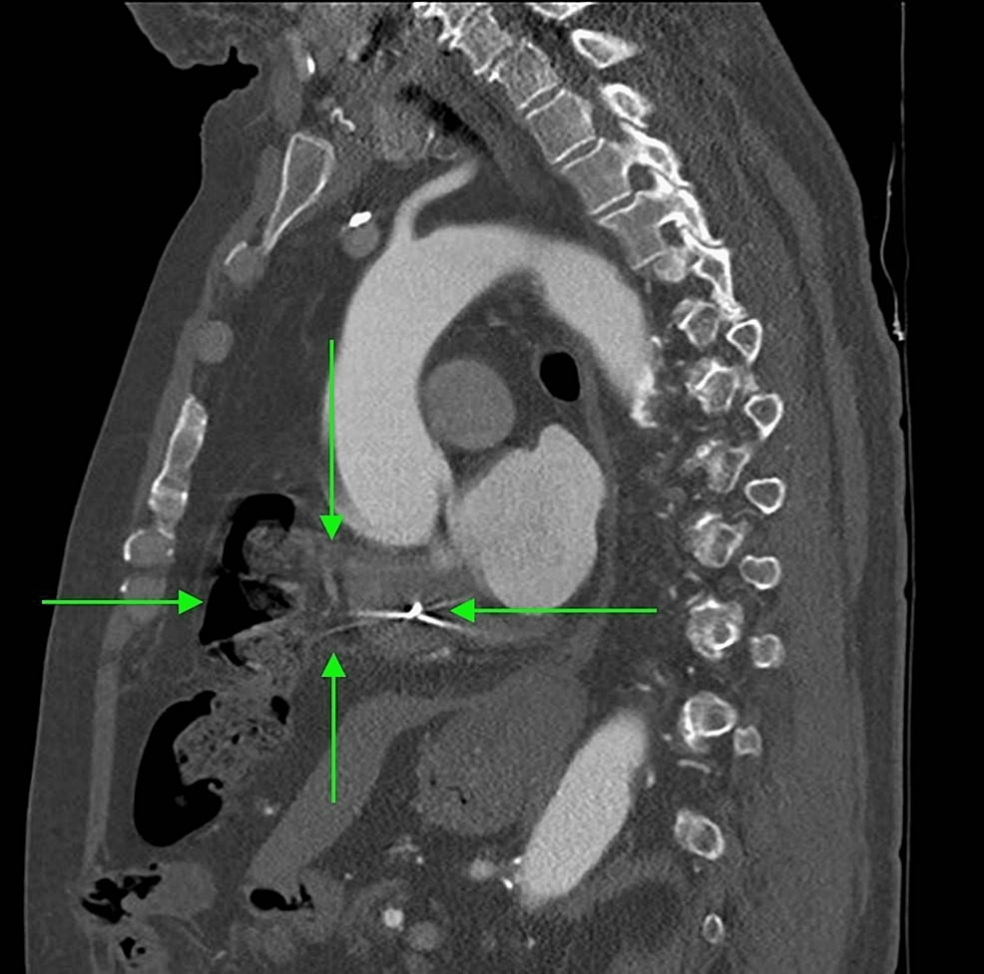
CTA of the chest revealing diaphragmatic herniation CTA: computed tomography angiogram.

**Figure 2:**
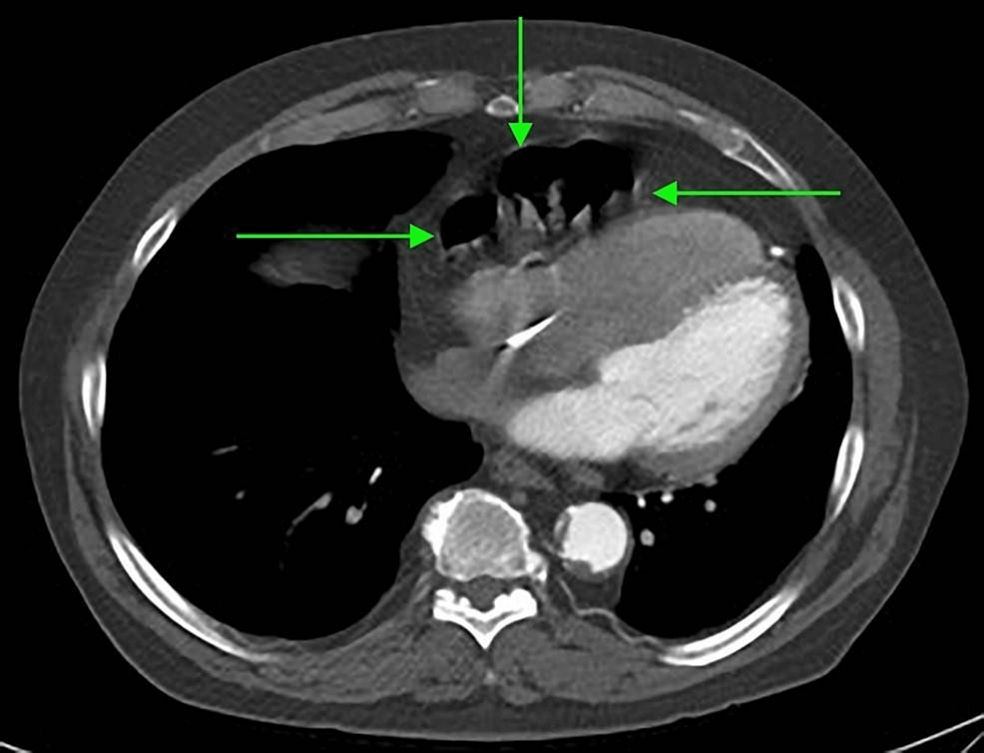
CTA of the chest, horizontal sections revealing diaphragmatic hernia CTA: computed tomography angiogram.

**Figure 3:**
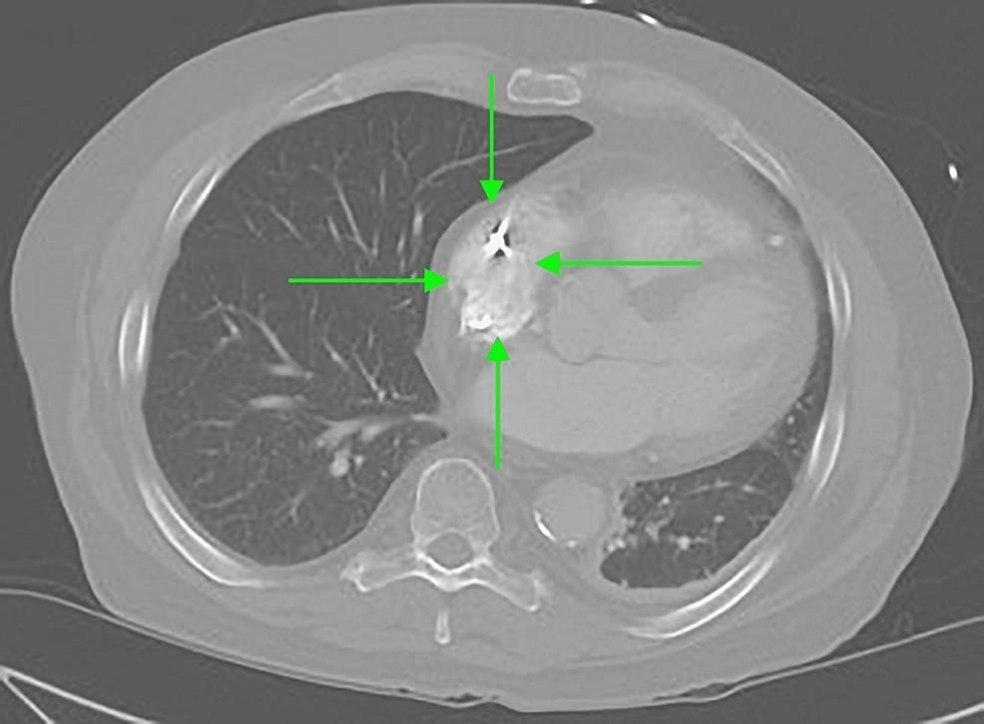
CTA of the chest, horizontal section, revealing small intra-pericardial hernia CTA: computed tomography angiogram.

**Figure 4:**
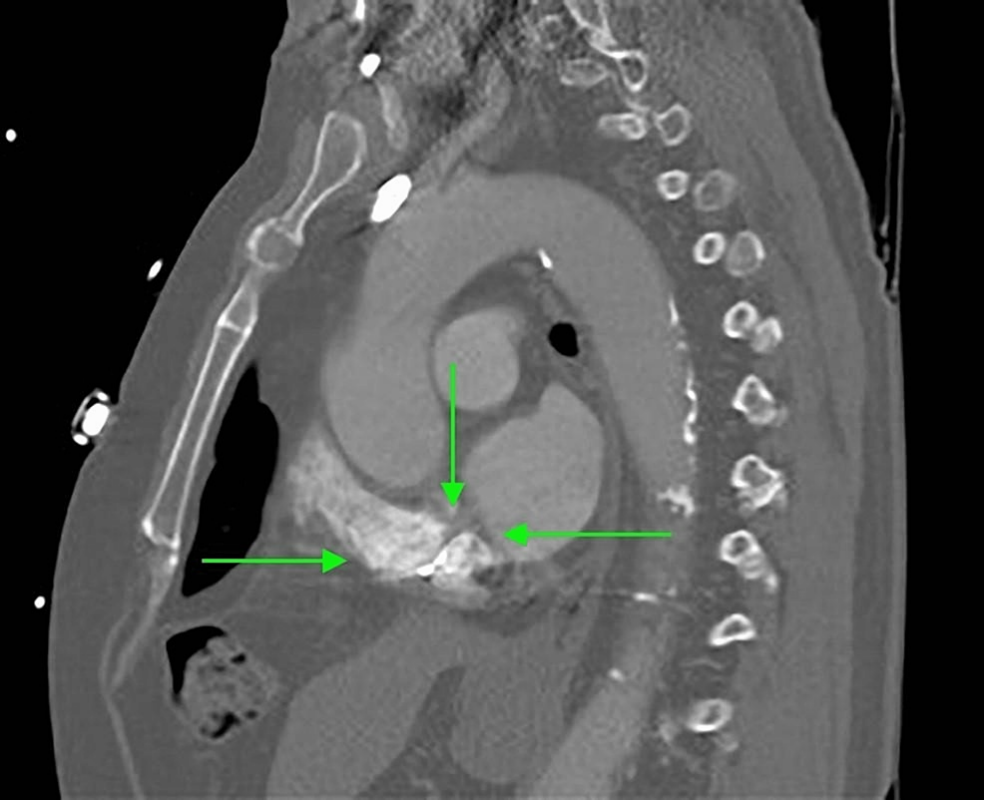
CTA of the chest, sagittal section, revealing small intra-pericardial hernia CTA: computed tomography angiogram.

Initial CTA Chest revealed cardiac enlargement, coronary artery disease, hilar and mediastinal lymphadenopathy, and minimal left lower lobe consolidation, around 2 weeks after the first surgical part of convergence (Figures 1, 2). Later on, a repeated CTA of the chest, more than three years after the convergence procedure, revealed a sinus of Valsalva 4.2 cm, a small hiatal hernia, and herniation of a segment of the transverse colon through a pericardial defect. The bowel is located within the pericardial fat (Figures 3, 4).

He was then referred to surgery for a hernia defect repair. The surgery was uncomplicated and on follow-up visits, he stated significant improvement in shortness of breath and denied any chest pain or pressure.

## Discussion

A pericardial-diaphragmatic hernia is the protrusion of abdominal viscera, such as the intestines, colon, or stomach, into the thorax, ending up in the pericardial cavity through the central tendon of the diaphragm into the pericardial sac [1].

Intrapericardial diaphragmatic hernia (IPDH) can be congenital or acquired. The congenital form of IPDH is extremely rare [2-3]. According to Shizas et al., the leading etiology of IPDH among 85 patients was trauma (56.5%), followed by iatrogenic interventions (30.6%). The most common herniated organs were the transverse colon (49.4%) and the greater omentum (48.2%) [11].

Iatrogenic defects commonly occur following trans-diaphragmatic creation of a pericardial window when treating conditions such as epicardial ablation procedures for AF, drainage of pericardial effusion, esophagectomy, coronary artery bypass graft (CABG) surgery, and trans-diaphragmatic resection of an inferior vena cava (IVC) neoplastic thrombus [5-10,12].

The clinical presentation and acuteness of IPDH are immensely variable, which makes clinical diagnosis challenging. Typical presenting symptoms include cardiorespiratory or gastrointestinal symptoms such as chest or abdominal pain, intestinal obstruction, cardiac tamponade, fatigue, exercise intolerance, or shortness of breath [13]. Therefore, IPDH should be suspected if any of these symptoms are present following surgical procedures involving the diaphragm. Imaging is key to the diagnosis, and CT of the chest is particularly helpful in this regard [11,14,15]. The treatment for IPDH is typically urgent surgical repair with mesh, either by a laparoscopic approach or by robotic technology [16,17].

## Conclusions

IPDH is quite a rare variant of diaphragmatic hernia, and it can occur as a complication of certain cardiac interventions such as CABG and convergence procedures. Due to the subtlety of the presentation, IDPH can be difficult to diagnose. Therefore, we recommend using a sub-xiphoid approach when performing convergence, rather than a diaphragmatic approach, to avoid the possibility of IPDH. Additionally, all medical professionals should be encouraged to report such cases to highlight the prevalence of this diagnosis.
